# Gait Initiation in Children with Rett Syndrome

**DOI:** 10.1371/journal.pone.0092736

**Published:** 2014-04-17

**Authors:** Ioannis Ugo Isaias, Mariangela Dipaola, Marlies Michi, Alberto Marzegan, Jens Volkmann, Marina L. Rodocanachi Roidi, Carlo Albino Frigo, Paolo Cavallari

**Affiliations:** 1 Department of Neurology, University Hospital Würzburg, Würzburg, Germany; 2 Department of Pathophysiology and Transplantation, LAMB Pierfranco & Luisa Mariani, University of Milan, Milan, Italy; 3 Department of Electronic, Information and Bioengineering, Biomedical Technology Laboratory, TBM Lab, Politecnico di Milano, Milan, Italy; 4 Fondazione Don Carlo Gnocchi IRCSS, Milan, Italy; University of California, San Diego, United States of America

## Abstract

Rett syndrome is an X-linked neurodevelopmental condition mainly characterized by loss of spoken language and a regression of purposeful hand use, with the development of distinctive hand stereotypies, and gait abnormalities. Gait initiation is the transition from quiet stance to steady-state condition of walking. The associated motor program seems to be centrally mediated and includes preparatory adjustments prior to any apparent voluntary movement of the lower limbs. Anticipatory postural adjustments contribute to postural stability and to create the propulsive forces necessary to reach steady-state gait at a predefined velocity and may be indicative of the effectiveness of the feedforward control of gait. In this study, we examined anticipatory postural adjustments associated with gait initiation in eleven girls with Rett syndrome and ten healthy subjects. Muscle activity (tibialis anterior and soleus muscles), ground reaction forces and body kinematic were recorded. Children with Rett syndrome showed a distinctive impairment in temporal organization of all phases of the anticipatory postural adjustments. The lack of appropriate temporal scaling resulted in a diminished impulse to move forward, documented by an impairment in several parameters describing the efficiency of gait start: length and velocity of the first step, magnitude and orientation of centre of pressure-centre of mass vector at the instant of (swing-)toe off. These findings were related to an abnormal muscular activation pattern mainly characterized by a disruption of the synergistic activity of antagonistic pairs of postural muscles. This study showed that girls with Rett syndrome lack accurate tuning of feedforward control of gait.

## Introduction

Rett syndrome (RTT) is an X-linked neurodevelopmental condition. Mutations in the gene encoding Methyl-CpG-binding protein 2 (MECP2) can be found in 95 to 97% of individuals with typical RTT [1], [2]. The clinical picture is defined by loss of spoken language and a regression of purposeful hand use, with the development of distinctive hand stereotypies and gait abnormalities. Epilepsy, breathing irregularities and gastro-intestinal problems may be also present [3]–[6]. At a clinical level, gait in children with RTT is characterized by ataxia, apraxia and spasticity with and without clonus. Affected girls develop a preference for one leg, putting it forward at every step as the foremost leg, using the contralateral one just for support and balance [7]. Based on available data, 20–40% children with RTT will never be able to walk. Furthermore, of the girls who gain the ability to walk, up to 80% might lose it along with disease progression [5], [8]. Despite being one of the most life burdening symptoms, detailed data on motor derangements and locomotion in RTT are not available.

**Figure 1:**
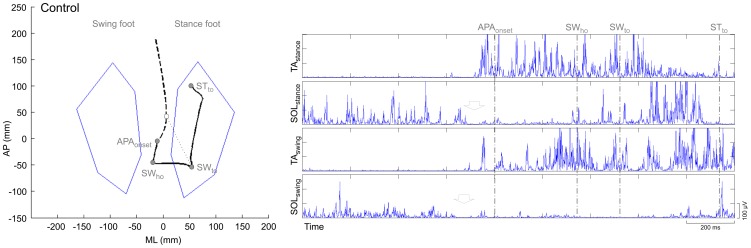
Recorded data at gait initiation. Centre of pressure (CoP, black line) and centre of mass (CoM, dashed line) displacement in a healthy subject (figure 1 left) and child with RTT (figure 2 left) with corresponding EMG activity of tibialis anterior (TA) and soleus muscles (SOL) of swing and stance foot (figure 1 right and 2 right). The dotted line (in figure 1 left and 2 left) shows CoP-CoM distance at toe-off of the swing foot (SWto). In figure 1 (right), arrows indicate bilateral suppression of the tonic activity of SOL which, together with the subsequent activation of TA, is responsible for the backward displacement of the CoP. This synergistic activity of pairs of postural muscles (i.e. TA and SOL) is not present in RTT girls (figure 2 right). *Imbalance phase*, from the instant APAonset, at which the CoP start moving backward, to the instant of heel-off of the swing foot (SWho). *Unloading phase*, from SWho to toe-off of the swing foot (SWto). STto is the instant of toe-off of the stance foot.

Gait initiation is the transition from quiet stance to steady-state condition of walking. The associated motor program seems to be centrally mediated [9]–[11] and includes preparatory adjustments (Anticipatory Postural Adjustments, APA) prior to any apparent voluntary movement of the lower limbs [11], [12]. APA contribute to postural stability [13] and to create the propulsive forces necessary to reach steady-state gait at a predefined velocity [14], [15]. These propulsive forces are generated by producing a misalignment between the centre of pressure (CoP), which is the point in which the resultant ground reaction force is applied at the foot-ground interface, and the vertical projection of the body centre of mass onto the ground (CoM) [14]. The distance between these two points in the sagittal plane can be modulated by changing the activity of the ankle plantarflexion/dorsiflexion muscles, while in the frontal plane the hip adduction/abduction muscle play a major role, associated with the activity of inversion/eversion muscles at the ankle [16], [17]. The APA phase can be subdivided into two sub-phases called imbalance phase and unloading phase. During the imbalance phase, the CoP moves backwards and towards the swing (leading) foot to produce a forward acceleration of the CoM directed also towards the stance (trailing) limb [14], [18], [19]. During the unloading phase, the CoP moves toward the stance foot, so that the body weight can be supported on this side and the swing foot can clear the ground to execute the first step. At this time the CoM is in front of the CoP and a propulsion force is produced by contracting the calf muscles. As a consequence, the CoP moves forward along a typical trajectory (figure 1 left) [11], [14], [18], [20], [21]. In healthy subjects, the CoP displacement during APA is associated with a typical electromyographic sequence consisting in the reduction, mono- or bilateral, of the activity in the soleus muscles (SOL) normally present during standing, followed by activation of the tibialis anterior muscles (TA) (figure 1 right) [11].

The study of gait initiation can help understanding the control of balance [22] and, in this perspective, the APA may be indicative of the effectiveness of the feedforward control of gait [23]. Indeed, it is only when the child is able to display systematic anticipatory behavior that it starts organizing it in relation to the characteristics of the velocity [24]. Given the great difficulties in obtaining the execution of a task on command in children with RTT, gait initiation might be the only available task to investigate locomotor behavior in these children.

## Patients and Methods

### Subjects

Eighteen children with RTT participated in the study. Despite enrolling children that were anamnestically able to maintain an upright position and walk unassisted, only eleven children (mean age 9±3 years) were able to complete a gait initiation task. In particular, some girls were unable to walk barefoot, others were anxious and disoriented when left alone. Often, RTT girls made a lateral or back step, possibly to gain a more stable standing position, before start walking.

Ten healthy children (HC; 4 females and 6 males; mean age 10±3 years) served as control group. No difference in biomechanic measurements of gait initiation between healthy male and female children was described.

Clinical evaluation was performed according to the Rett Assessment Rating Scale (RARS) (table 1). The scale evaluates the specific phenotypic characteristics of RTT syndrome in five different domains (i.e. cognitive, sensory, motor, emotional, behavioral). Each of the 31 items is rated on a 4-point scale, where 1 = within normal limits, 2 = infrequent or low abnormality, 3 = frequent or medium-high abnormality, and 4 = strong abnormality. Intermediate ratings are also possible. A total score is computed by summing the ratings of all 31 items. The RARS was established by a standardization procedure, involving a sample of 220 subjects, and proved being a valid and reliable instrument to identify the level of severity of RTT syndrome [25].

**Table 1:** Demographic and clinical characteristics.

Patient n.	1	2	3	4	5	6	7	8	9	10	11
**Genetic mutation**	c.1327G>A	c.1156_1197del41	c.916C>T	c.763C>T	c.602_603insGGCC	c.455C>G	c.431delA	c.397C>T	c.1156del41	c.1052_188del37	c.808C>T
**Age**	10	6	7	7	7	15	7	7	12	8	15
**RARS – Cognition**	19	8	8	10.5	8.5	14	7	7	9	8	16
**RARS – Sensory**	2	2	2	2	2	2	2	2	2	2	4
**RARS – Motor**	7.5	4	4	6	5	7	5	5	5	4.5	7
**RARS – Emotion**	4.5	2	2	2	2	4	2	2	3	2.5	4
**RARS – Indipendent activities**	10	5	5	5	5	6	5.5	6	7	6	11
**RARS – Typical features**	30.5	21	19	17	18	24	23	21.5	23	21	33
**RARS – Total score**	73.5	42	40	42.5	40.5	57	44.5	43.5	49	44	75
**Age of acquisition of independent gait (ms)**	18	19	30	15	18	16	12	18	20	24	20
**Age of appearance of stereotypies (ms)**	12	39	24	30	24	40	24	18	9	36	18
**Spasticity**	No	No	No	No	No	LL-LR (mild)	No	LL-LR (mild)	No	No	UL-LL-LR (mild)
**Dystonia**	No	No	No	No	No	LL (mild)	No	No	No	LL-LR (mild)	No
**Scoliosis**	Mild	No	No	Mild	No	Mild	No	No	Moderate	Moderate	Mild
**Microcephaly at the time of observation**	No	No	Yes	Yes	Yes	No	Yes	No	Yes	Yes	Yes
**Epilepsy**	Yes	Yes	Yes	Yes	No	Yes	No	Yes	Yes	No	Yes

All patients were found to have mutations of deletion in the MECP2 gene.

The local institutional review board (Section of Human Physiology, Department of Pathophysiology and Transplantation) approved the study and all parents (or guardians) provided written informed consent. Data are freely available upon request. Confidentiality rules related to human-subject research will apply.

### Experimental protocol

Subjects were placed over a force platform upright, with feet parallel, and asked to start walking spontaneously as soon as they received a verbal cue; RTT children were called repeatedly by the parents seated at about 5 m distance in front of them. All children were not supported nor helped at any time during the gait initiation task. We evaluated only tasks when at least two strides after gait start were performed; this assured us that RTT children had elaborated a real intention to start walking.

### Recording system

Movement kinematics were recorded using an optoelectronic system (SMART 1.10, BTS, Italy), consisting of six video cameras working at a sampling rate of 60 Hz and located around a calibrated volume of 5×3×2 m3. A full body markers dataset (29 retro-reflective markers with 12 mm diameter – LAMB protocol) [26] was used to analyze the motion of head, trunk, upper limbs and lower limbs in order to compute the global CoM. Anthropometric parameters of each subject were used for the estimation of internal joint centers. These, in turn, enabled us to calculate head, trunk, and lower limbs kinematics. Anthropometric data were obtained from Zatsiorski regression equations [27]. All recorded data were visually inspected, and trials performed incorrectly were rejected. Two specific sets of parameters were automatically extracted by using ad hoc algorithms.

Ground reaction forces and CoP position was obtained by means of a dynamometric platform (KISTLER 9286A, Winterhur, Switzerland) embedded in the floor (sampling rate 960 Hz).

Surface electromyographic (EMG) activity, during gait initiation trials, was recorded using a telemetric eight-channel system (TELEMG, BTS, Milan, Italy) from TA and SOL muscles of both legs. Myoelectric signals were collected by preamplified Ag/AgCl electrodes (diameter: 25 mm, bipolar configuration, inter-electrode distance: 20 mm), band-pass filtered (*f*_high-pass_ = 10 Hz, *f*_low-pass_ = 200 Hz) and acquired at a sampling frequency of 960 Hz and a resolution of 16 bit. A digital zero-phase shift eight-order Butterworth high-pass filter with a cutoff frequency of 20 Hz was applied in order to remove movement artifacts and finally the signals were rectified.

### Events identification and parameters evaluated

The temporal transition between quiet standing and APAonset was defined as the first sample point in the CoP trajectory at which the CoP started moving backward and towards the swing limb (figure 1 left) [28].

SWho (heel-off of the swing foot) is the most lateral motion of the CoP towards the swing foot. SWto (toe-off of the swing foot) is the event when CoP shifts from lateral to anterior motion. STto (toe-off of the stance limb) is the event corresponding to the time when the stance limb breaks contact with the supporting surface [28], [29].

The parameters extracted to describe the APA phases were: 1) the duration of imbalance (from APAonset to SWho) and unloading phases (from SWho to SWto), 2) the antero-posterior (AP) and 3) medio-lateral (ML) shift of the CoP (normalized to the foot length, measured as the distance between lateral malleolar and fifth metatarsal markers), 4) the CoP length and 5) mean velocity. In addition, to evaluate the efficiency of gait start, we described the following parameters: 6) the magnitude of the CoP-CoM vector at SWto (i.e. CoP-CoM distance at SWto); 7) the orientation of the CoP-CoM vector with respect to the progression line at SWto; 8) the length and 9) the velocity of the first step. Spatial parameters were normalized on the basis of body height (%BH).

### CoP–CoM vector magnitude and orientation

The CoP and CoM trajectory data were exported and time-synchronized with a frequency of 60 Hz. The superimposition of CoP and CoM in the ground level plane was obtained by removing the respective average values computed in the steady state time window, before any occurrence of voluntary movement. With a common time base and a common spatial origin, the quantity CoP-CoM distance was easily determined by applying a conventional geometric distance formula between the coordinates of CoP and the coordinates of CoM at distinct points in time t_i_:
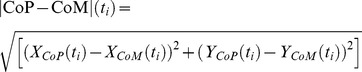
where 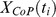 was the displacement of the CoP in AP direction at time t_i_, 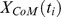 was the displacement of the CoM in AP direction at time t_i_, and 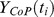 and 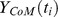 are the corresponding values for the ML direction.

The orientation of the CoP-CoM vector was calculated with respect to the progression line at SWto. The vector joining CoP and CoM represents the direction of CoM acceleration according to the inverted pendulum model [17].

### Length and velocity of first step

The length of the first step was defined as the measure of the anterior displacement of the ankle marker of the swing foot, from the initial standing position to next heel strike. The velocity of the first step was defined as the maximum value of the time derivative of the ankle marker displacement.

### Data analysis

For each subject, variables were averaged over the trials (at least three for all subjects) of each test. Considering that data were not normally distributed, the differences between HC and RTT groups were analyzed non-parametrically by using the Wilcoxon-Mann-Whitney U Test. Spearman's rho was applied to investigate statistical dependence among APA variables and parameters describing the efficiency of gait start (see above). Level of significance was set to 0.05.

## Results

**Figure 2:**
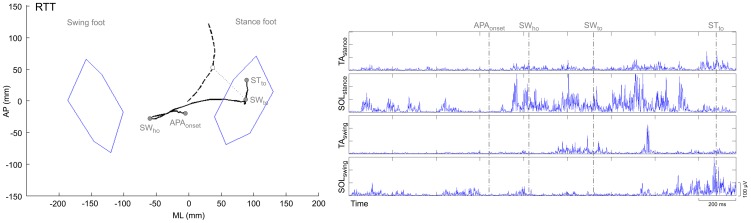
Recorded data at gait initiation. Centre of pressure (CoP, black line) and centre of mass (CoM, dashed line) displacement in a healthy subject (figure 1 left) and child with RTT (figure 2 left) with corresponding EMG activity of tibialis anterior (TA) and soleus muscles (SOL) of swing and stance foot (figure 1 right and 2 right). The dotted line (in figure 1 left and 2 left) shows CoP-CoM distance at toe-off of the swing foot (SWto). In figure 1 (right), arrows indicate bilateral suppression of the tonic activity of SOL which, together with the subsequent activation of TA, is responsible for the backward displacement of the CoP. This synergistic activity of pairs of postural muscles (i.e. TA and SOL) is not present in RTT girls (figure 2 right). *Imbalance phase*, from the instant APAonset, at which the CoP start moving backward, to the instant of heel-off of the swing foot (SWho). *Unloading phase*, from SWho to toe-off of the swing foot (SWto). STto is the instant of toe-off of the stance foot.

Kinematic measurements are listed in table 2. Of relevance, in children with RTT a selective impairment of the velocity of CoP displacement was described during both the imbalance and unloading phase. Interestingly, besides AP displacement of the CoP during the unloading phase, spatial positioning as well as length of the CoP during APA was within the normal range (figure 2 left). Indeed, CoP-CoM distance at SWto was normal in girls with RTT, but not the velocity of CoM at SWto nor the orientation of the CoP-CoM vector. Lack of appropriate temporal scaling in RTT resulted in reduced impulse to move forward, as described by a reduction of length and velocity of the first step.

**Table 2:** Kinematic measurements.

	RTT		Healthy subjects		
	Median	Range	Median	Range	p value
Imb. phase duration (s)	0.47	0.13–0.51	0.37	0.24–0.46	0.36
Imb. phase AP displacement of CoP (%FL)	8.11	1.84–16.99	14.45	7.92–20.87	0.59
Imb. phase ML displacement of CoP (%FL)	9.82	2.69–35.36	13.03	6.7–23.57	0.23
Imb. phase CoP length (mm)	42.23	5.64–148.85	51.84	36.91–86.15	0.94
Imb. phase CoP mean velocity (mm/s)	87.16	21.60–137.43	127.42	103.23–279.54	0.01
Unl. phase duration (s)	0.81	0.61–1.12	0.33	0.24–0.59	0.006
Unl. phase AP displacement of CoP (%FL)	25.26	12.32–36.51	10.66	4.81–14.08	0.0004
Unl. phase ML displacement of CoP (%FL)	68.03	12.62–119.84	53.23	29.63–59.53	0.1385
Unl. phase CoP length (mm)	179.08	23.01–242.03	136.25	79.6–170.74	0.3671
Unl. phase CoP mean velocity (mm/s)	176.82	67.54–459.55	334.78	282.68–648.59	0.0056
Magnitude of CoP-CoM vector at SWto (%BH)	6.44	2.5–8.91	5.45	3.7–7.73	0.15
Orientation of CoP-CoM vector (deg) with respect to the progression line (at SWto)	48.66	20.4–71.73	30.41	22.04–33.77	0.02
Length of the first step (%BH)	19.8	11.35–40.5	31.13	27.25–32.85	0.04
Velocity of the first step (mm/s)	670.12	657.06–1115.70	1662.40	1458.35–1770.00	0.001

AP = Antero-posterior; ML = Medio-materal. Imb. = Imbalance; Unl. = Unloading; FL = foot length; BH = body height; SWto = Heel-off of the swing foot. p values refers to Wilcoxon-Mann-Whitney U Test.

EMG recordings are listed in table 3. A lack of synergistic activity of SOL and TA was observed in all trials of RTT children. In particular, the burst of TA was reduced in amplitude, delayed and desynchronized (figure 2 right).

**Table 3:** EMG recordings (RMS).

	RTT		Healthy subjects		
	Median	Range	Median	Range	p value
Imb. phase SOL stance foot	1.31	0.23–3.55	0.79	0.47–1.03	0.2135
Imb. phase TA stance foot	0.91	0.54–1.81	19.11	9.27–102.04	0.0004
Imb. phase SOL swing foot	0.78	0.41–2.83	0.95	0.6–1.56	0.8065
Imb. phase TA swing foot	0.9	0.33–2.79	20.91	5.97–33.25	0.0002
Unl. phase SOL stance foot	1.78	1.15–8.57	1.71	0.79–3.18	0.4772
Unl. phase TA stance foot	1.88	0.43–2.78	18.69	4.15–125.27	0.0005
Unl. phase SOL swing foot	1.64	0.46–5.78	0.93	0.51–1.46	0.1053
Unl. phase TA swing foot	2.47	0.57–10.52	17.13	8.80–42.88	0.0001

Imb. = Imbalance; Unl. = Unloading; SOL = Soleus muscle; TA = Tibialis anterior muscle; p values refers to Wilcoxon-Mann-Whitney U Test.

Several correlations were found in the HC cohort among the kinematic measurements of gait start efficiency. In particular, in the imbalance phase, AP displacement of CoP correlated with instant AP velocity of CoM at SWto (ρ = 0.78, p = 0.003) and CoP-CoM distance at SWto (ρ = 0.82, p = 0.023). In the unloading phase, ML displacement of CoP correlated with the CoP-CoM vector orientation (ρ = 0.82, p = 0.036). None of these correlations resulted statistically significant in the RTT cohort. On the contrary, in RTT children the length of the first step correlated with ML displacement and velocity of CoP, in the unloading phase (ρ = 0.75, p = 0.052; ρ = 0.85, p = 0.013, respectively). Also in this cohort, velocity of first step correlated with CoP-CoM distance at SWto (ρ = 0.92, p = 0.002).

## Discussion

In children with RTT, investigation of APA revealed a distinctive impairment in timing rather than the spatial organization of gait initiation, particularly in the unloading phase. These findings may be related to an abnormal muscular activation pattern characterized by asynergistic activity of pairs of postural muscles (i.e. TA and SOL).

The deactivation of the plantarflexors, but more importantly the activation of the TA, [11] is responsible for the backward shift of the CoP which normally promotes the initial forward leaning of the body. The anticipatory behavior of the TA is an index of anticipatory scaling or feedforward control of movement [30], [31] and it is a prerequisite for the emergence of preparatory adjustments during gait initiation.

Girls with RTT showed a pattern similar to children with age of 4-year or below characterized, in the imbalance phase, by a predominant ML displacement of CoP and a low TA burst [32]. It is possible that children with RTT needed a greater effort to produce the disequilibrium required to initiate the single limb support phase. To simplify this task, RTT girls might produce such a disequilibrium predominantly in the ML direction, as this plane of movement involves fewer degrees of freedom [33]. A wider base of support, often seen in RTT girls, may also promote ML displacement by reducing the pelvic rotation that allows for rotatory movements of the trunk about the vertical axis [34]–[36]. Of relevance, in RTT children the length of the first step correlated selectively with ML displacement in the unloading phase.

The magnitude of the initial TA burst, and concomitant backward shift of CoP, are normally associated with the forthcoming gait velocity [11]. In girls with RTT, the lack of the anticipatory activation of the TA may suggest that the control mechanisms of gait initiation are not integrated into a locomotor program. RTT children would be unable to adjust the magnitude of their anticipatory responses as a possible result of an impaired representation of their forthcoming velocity. It is worth mentioning that, as for spatial organization of APA, mastering of spatio-temporal characteristics of gait (e.g. tuning backward shift to desired forthcoming velocity), is already completed at the age of 6-year [24], [37]. Failure to develop a TA burst could also result from either a less efficient descending motor command (motor unit recruitment) or from immature muscle properties. However, both factors appear unlikely, since all children were able to develop a large phasic TA burst during the swing phase of the first three strides (data not shown).

We cannot exclude that abnormal standing posture, possibly related to dystonia or spasticity in few girls (table 1), could have been partly responsible for the observed changes in the APA phases. However, this would have influenced also spatial parameters of APA, which resulted within the normal range in our RTT cohort. Of relevance, in this study we only took into consideration recordings where children with RTT had a base of support similar to controls. Still, although in some cases imposed, it was not possible to standardize the base of support and corresponded of the preferred (natural) stance of the children. However, differences of anthropometric characteristic [32], [38] or posture (e.g. forward leaning) [11] were reported not to account for a reduction of the initial TA burst and the concomitant smaller backward shift of the CoP.

Reduced TA activity, especially of the swing foot, has been previously described in hemiplegic children with cerebral palsy (CP) [39] and interpreted as anticipatory planning deficit, which may be caused by an impairment at the motor imagery level [40]. However, a direct comparison with our findings may not be possible since children with hemiplegic CP demonstrated a tendency to shift the CoP towards the less affected limb, which is usually their stance limb. This shift may determine a reduced TA activity of the swing foot as a results of an increased reliance upon the stance limb to create the posterior shift of the CoP and redirect the ground reaction force vector anteriorly to facilitate forward motion [39].

Taken together, our results suggest that girls with RTT might lack accurate tuning of feedforward control of movements at gait initiation. These findings may be relevant with regards to rehabilitation practice. Guidelines for motor rehabilitation in RTT consider intensely applied physical therapy [41]. Indeed, there is preliminary evidence of a protective effect of learning to walk on the risk of fracture in children with RTT [42]. Balance exercises, eye/feet coordination, walking on different surfaces and crossing obstacles are some of the goals in motor rehabilitation. A specific training on gait initiation and on practices to facilitate tuning of feedforward control of movements has never been described in motor rehabilitation protocols for RTT girls. Our preliminary results also suggest that motor intervention in girls with RTT should include preparatory adjustments of gait initiation inducing backward disequilibrium reactions and strengthening a TA activation in order to promote the initial forward leaning of the body which is necessary to initiate walking. Further research should be encouraged to obtain evidence based protocols of intervention, such as intensive models of motor intervention for short periods [43], [44], and to investigate the relationships between specific motor exercises and quality of gait in RTT children.

## References

[1] Amir RE, Van den Veyver IB, Wan M, Tran CQ, Francke U, et al (1999) Rett syndrome is caused by mutations in X-linked MECP2, encoding methyl-CpG- binding protein 2. Nat Genet 23: 185–188. 10508514 10.1038/13810

[2] Neul JL, Fang P, Barrish J, Lane J, Caeg EB, et al (2008) Specific mutations in methyl- CpG-binding protein 2 confer different severity in Rett syndrome. Neurology 70: 1313–1321. 18337588 10.1212/01.wnl.0000291011.54508.aa PMC2677974

[3] Rett A (1966) On a unusual brain atrophy syndrome in hyperammonemia in childhood. Wien Med Wochenschr 116: 723–726. 5300597

[4] Hagberg B, Aicardi J, Dias K, Ramos O (1983) A progressive syndrome of autism, dementia, ataxia, and loss of purposeful hand use in girls: Rett's syndrome: report of 35 cases. Ann Neurol 14: 471–479. 6638958 10.1002/ana.410140412

[5] Witt Engerström I (1990) Rett syndrome in Sweden. Neurodevelopment–disability–pathophysiology. Acta Paediatr Scand Suppl 369: 1–60. 1701067

[6] Neul JL, Kaufmann WE, Glaze DG, Christodoulou J, Clarke AJ, et al (2010) Rett Syndrome: Revised Diagnostic Criteria and Nomenclature. Ann Neurol 68: 944–950. 21154482 10.1002/ana.22124 PMC3058521

[7] Dan B, Cheron B (2008) Postural control in children with Rett syndrome or Angelman syndrome. In: Hadders-Algra M, Brogren Carlberg E, Eds. Posture: A Key Issue in Developmental Disorders. London: Mac Keith Press. pp. 148–169.

[8] Colvin L, Fyfe S, Leonard S, Schiavello T, Ellaway C, et al (2003) Describing the phenotype in Rett syndrome using a population database. Arch Dis Child 88: 38–43. 12495959 10.1136/adc.88.1.38 PMC1719276

[9] Das P, McCollum G (1988) Invariant structure in locomotion. Neuroscience 25: 1023–1034. 3043253 10.1016/0306-4522(88)90055-3

[10] Forssberg H (1985) Ontogeny of human locomotor control I. Infant stepping, supported locomotion and transition to independent locomotion. Exp Brain Res 57: 480–493. 3979491 10.1007/BF00237835

[11] Crenna P, Frigo C (1991) A motor programme for the initiation of forward-oriented movements in humans. J Physiol Lond 437: 635–653. 1890653 10.1113/jphysiol.1991.sp018616 PMC1180068

[12] Massion J (1992) Movement, posture and equilibrium: interaction and coordination. Progr Neurobiol 38: 35–56. 10.1016/0301-0082(92)90034-c 1736324

[13] McIlroy WE, Maki BE (1999) The control of lateral stability during rapid stepping reactions evoked by antero-posterior perturbation: does anticipatory control play a role. Gait Posture 9: 190–198. 10575080 10.1016/s0966-6362(99)00013-2

[14] Brenière Y, Do MC, Bouisset S (1987) Are dynamic phenomena prior to stepping essential to walking? J Mot Behav 19: 62–76. 23944913 10.1080/00222895.1987.10735400

[15] Lepers R, Breniere Y (1995) The role of anticipatory postural adjustments and gravity in gait initiation. Exp Brain Res 107: 118–124. 8751069 10.1007/BF00228023

[16] Winter DA (1995) Human balance and postural control during standing and walking. Gait Posture 3: 193–214.

[17] Winter DA, Patla AE, Ishac M, Gage WH (2003) Motor mechanisms of balance during quiet standing. J Electromyogr Kinesiol 13: 49–56. 12488086 10.1016/s1050-6411(02)00085-8

[18] Brenière Y, Do MC (1986) When and how does steady state gait movement induced from upright posture begin? J Biomech 19: 1035–1040. 3818673 10.1016/0021-9290(86)90120-x

[19] Jian Y, Winter DA, Ishac MG, Gilchrist L (1993) Trajectory of the body COG and COP during initiation and termination of gait. Gait Posture 1: 9–22.

[20] Mann RA, Hagy JL, White V, Liddell D (1979) The initiation of gait. J Bone Joint Surg 61: 232–239. 422607

[21] Nissan M, Whittle MW (1990) The initiation of gait in normal subjects: a preliminary study. J Biomed Eng 12: 165. 2319768 10.1016/0141-5425(90)90139-e

[22] Carlsoo A (1966) The initiation of walking. Acta Anatomica 65: 1–9. 5965964 10.1159/000142864

[23] Hayes KC, Riach CL (1990) Preparatory postural adjustements and postural sway in young children. In: Woollacott M, Horak F, edotors. Posture and gait: control mechanisms, vol II. Portland: University of Oregon Books. pp. 255–258.

[24] Ledebt A, Bril B, Brenière Y (1998) The build up of anticipatory behaviour: an analysis of the development of gait initiation in children. Exp Brain Res 120: 9–17. 9628398 10.1007/s002210050372

[25] Fabio RA, Martinazzoli C, Antonietti A (2005) Costruzione e standardizzazione dello strumento “R.A.R.S.” (Rett Assessment Rating Scale). Ciclo Evol Disabil 8: 257–281.

[26] Ferrari A, Benedetti MG, Pavan E, Frigo C, Bettinelli D, et al (2008) Quantitative comparison of five current protocols in gait analysis. Gait Posture 28: 207–216. 18206374 10.1016/j.gaitpost.2007.11.009

[27] Zatsiorsky V, Seluyanov V (1983) The mass and inertia characteristics of the main segments of the human body. In: H Matsui & K Kobayashi, Eds. Biomechanics VIII-B. Champaign, IL: Human Kinetics. pp. 1152–1159.

[28] Martin M, Shinberg M, Kuchibhatla M, Ray L, Carollo JJ, et al (2002) Gait initiation in community-dwelling adults with Parkinson disease: comparison with older and younger adults without the disease. Phys Ther 82: 566–577. 12036398

[29] Carpinella I, Crenna P, Calabrese E, Rabuffetti M, Mazzoleni P, et al (2007) Locomotor Function in the Early Stage of Parkinson's Disease. IEEE 15: 543–551. 10.1109/TNSRE.2007.908933 18198712

[30] Gahery Y (1987) Associated movements, postural adjustements and synergies: some comments about the historic and significance of the three motor concenpts. Arch Ital Biol 125: 345–360. 3326536

[31] Rosenbaum DA (1984) The planning and control of movements. In: Anderson JR, Kosslyn SM, Eds. Tutorials in learning and memory: essay in honor of Gordon. San Francisco: Freeman. pp. 219–233.

[32] Malouin F, Rchards CL (2000) Prepartory adjustements during gait initiation in 4–6-year-old children. Gait Posture 11: 239–253. 10802437 10.1016/s0966-6362(00)00051-5

[33] Berstein N (1967) Coordination and Regulation of Movement. New York: Pergamon.

[34] Forssberg H, Hirschfeld H, Stokes VP (1991) Development of human locomotor mechanisms. In: Shimamura M, Grillner S, Edgerton VR, Eds. Neurobiological Basis of Human Locomotion. Tokyo: Japan Scientific Society Press. pp. 259–273.

[35] Malouin F, Menier C, Comeau F, Dumas F, Richards CL, et al (1994) Dynamic Weight transfer during gait initiation in hemiparetic adults and effect of foot position. Soc Neurosci 241: 3.

[36] Malouin F, Richards CL, Trahan J, Menier C, Dumas F, et al (1996) Gait initiation in 4-6-year-old children. Soc Neurosci 802: 8.

[37] Brenière Y, Bril B, Fontaine R (1989) Analysis of the transition from upright stance to steady state locomotion in children with under 200 days of autonomous walking. J Mot Behav 21: 20–37. 15117670 10.1080/00222895.1989.10735462

[38] Caderby T, Dalleau G, Leroyer P, Bonazzi B, Chane-Teng D, et al (2013) Does an additional load modify the anticipatory postural adjustements in gait initiation? Gait Posture 37: 144–146. 22796245 10.1016/j.gaitpost.2012.06.012

[39] Stackhouse C, Shewokis PA, Pierce SR, Smith B, McCarthy J, et al (2007) Gait initiation in children with cerebral palsy. Gait Posture 26: 301–308. 17081756 10.1016/j.gaitpost.2006.09.076

[40] Mutsaarts M, Steenbergen B, Bekkering H (2005) Anticipatory planning of movement sequences in hemiparetic cerebral palsy. Motor Control 9: 439–458. 16333147 10.1123/mcj.9.4.439

[41] Lotan M, Hanks S (2006) Physical therapy intervention for individuals with Rett syndrome. ScientificWorldJournal 6: 1314–1338. 17041720 10.1100/tsw.2006.187 PMC5917408

[42] Downs J, Bebbington A, Woodhead H, Jacoby P, Jian L, et al (2008) Early determinants of fractures in Rett syndrome. Pediatrics 121: 540–546. 18310203 10.1542/peds.2007-1641

[43] Polovina S, Polovina TS, Polovina A, Polovina-Proloscić T (2010) Intensive rehabilitation in children with cerebral palsy: our view on the neuronal group selection theory. Coll Antropol 34: 981–988. 20977092

[44] Sorsdahl AB, Moe-Nilssen R, Kaale HK, Rieber J, Strand LI (2010) Change in motor basic abilities, quality of movement and daily activities following intensive, goal-directed, activity-focused physiotherapy in a group setting for children with cerebral palsy. BMC Pediatr 27: 10–26. 10.1186/1471-2431-10-26 PMC2878295 20423507

